# Indicators and risk factors of infectious laryngotracheitis in layer hen flocks in Algeria

**DOI:** 10.14202/vetworld.2021.182-189

**Published:** 2021-01-22

**Authors:** Omar Salhi, Chafik Redha Messaï, Nassim Ouchene, Iman Boussaadi, Hassiba Kentouche, Rachid Kaidi, Djamel Khelef

**Affiliations:** 1 Biotechnology Laboratory of Animal Reproduction, Institute of Veterinary Sciences, Blida, Algeria; 2 Laboratory of Research Health and Animal Production, High National Veterinary School, Algiers, Algeria

**Keywords:** Algeria, enzyme-linked immunosorbent assay, infectious laryngotracheitis, layer hens, vaccine

## Abstract

**Background and Aim:**

Since 2017, there have been epidemics with respiratory disorders in the laying hen farms in Algeria, as signs and lesions, respiratory difficulties, and hemorrhagic tracheitis, which closely like laryngotracheitis. This study aimed to analyze the epidemiological, serological, and clinical indicators, as well as the risk factors, of infectious laryngotracheitis (ILT) in layer hen flocks in Algeria.

**Materials and Methods:**

A total of 1728 layer hens were sampled randomly from 48 poultry houses. Blood samples were collected from each hen at the wing vein area, and an indirect enzyme-linked immunosorbent assay was done using an IDvet^®^ kit.

**Results:**

The flocks showed 56.25% seroprevalence. Clinical signs and gross lesions of ILT suspect cases included respiratory signs characterized by hemorrhagic tracheitis and sinusitis; conjunctivitis; egg drop; and a low mortality rate varying from 5% to 20%. Statistical analyses showed the effect of risk factors on the seropositivity for ILT in 48 layer flocks. When the vaccination was not applied, flocks were significantly more seropositive by 54% (odds ratio OR=1.54, p=0.01) compared to vaccinated flocks. Furthermore, flocks with poor hygiene were more seropositive by 68% (OR=1.68, p=0.002) compared to those with good hygiene. Finally, flocks with decreased egg production between 10% and 30% were significantly more seropositive by 42% (OR=1.42, p=0.04) than those with egg production >30%.

**Conclusion:**

The serological survey revealed anti-ILT virus antibodies, signifying the circulation of this virus in layer hen farms in Algeria. Correct vaccination protocol, strict biosecurity measures, rapid diagnosis, and detection of latent carriers are necessary to control and eradicate the disease from layer farms.

## Introduction

Infectious laryngotracheitis (ILT) is a highly contagious respiratory viral disease in chickens. It causes considerable economic losses due to the resulting steep declines in egg laying as well as its high mortality rates. The ILT virus (ILTV) belongs to the genus *Iltovirus*, family Herpesviridae, and subfamily Alphaherpesvirinae [1,2].

The disease manifests in two forms: A severe epizootic form and a mild enzootic form. The severe infection manifests in respiratory symptoms such as respiratory depression, gasping, hemorrhagic tracheitis, and bloody mucus sputum, and it has a high rate of mortality. The mild enzootic form is more present in developed poultry industries, and presents with varied clinical expressions, including mucoid tracheitis, sinusitis, conjunctivitis, general unthriftiness, and a low rate of mortality [3,4]. Risk mitigation factors, such as sanitary barriers and hygienic measures, play an important role in the gravity of viral diseases in affected farms [5,6]. In fact, the high contagiousness of ILT is due to the easy transmission and propagation of the virus, facilitated by sick chickens and fomites, lack of biosecurity measures, movement of affected animals, and the improper disposal of contaminated litter [4].

The signs of this respiratory disease are not evocative, even when tracheal plugs with mucosal hemorrhage are observed [4]. While gross lesions and clinical signs in sick birds can help to diagnose the disease, laboratory confirmation of the disease is required [7]. Histopathology and real-time PCR methods are the two techniques strongly recommended for this disease. In addition, the enzyme-linked immunosorbent assay (ELISA) is useful for the diagnosis of viral diseases [8,9]. ELISA was found to be a sensitive method to measure anti-ILTV antibodies, and this technique is often used for its advantages [4].

Since 2017, Algeria has remained a country free from the disease. However, we are witnessing clinical signs in the field that is very similar to the description of ILT. In response, the ILT vaccine was used and added to the vaccination protocol for laying hens in Algeria.

The objective of this study was to illuminate the circulation of ILTV on layer hens flocks in Algeria. Epidemiological survey, clinical signs, postmortem lesions, and serological tests (ELISA) were done to evaluate the risk factors associated with the ILT in farms with suspected viral infections.

## Materials and Methods

### Ethical approval

The experiments were carried out according to the recommendations of the Institutional Committee for the Protection of Animals of the National Administration of Higher Education and Scientific Research of Algeria (98-11, Act of August 22, 1998).

### Study period and location

**Figure-1:**
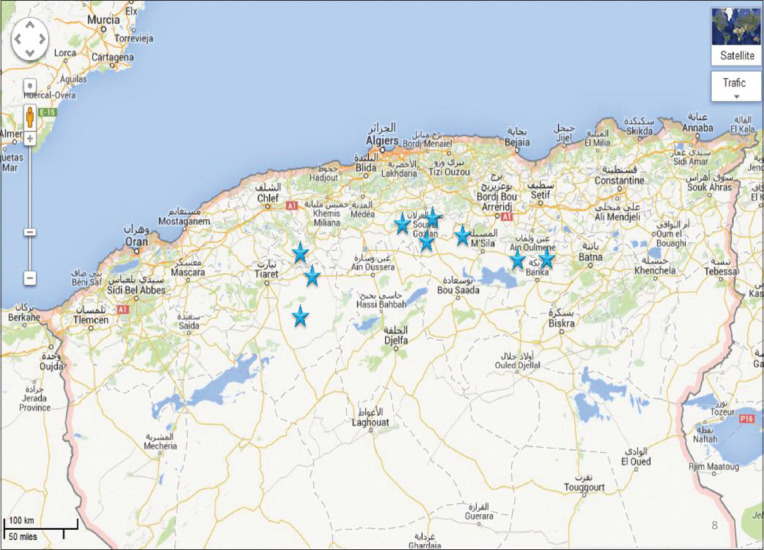
Map of the 48 laying hen farms covered by the serological study of laryngotracheitis in Algeria. [Source: google.dz/maps].

We conducted our study from June 2018 to January 2020, using samples collected from 48 commercial layer hen flocks comprising four different strains (ISA Brown, Tetra-SL, Lohman Tradition, Hy-line) in Algeria (longitude 36° and latitude 3°; Figure-1). Layers were between 22 and 65 weeks of age, and the farms contained between 5000 and 100,000 birds/farm. Flocks were selected based on indicators of ILT infection within the flock, as discussed in the section on clinical diagnosis.

### Experimental design

The study was carried out on flocks suspected of ILT based on clinical signs, gross lesions, and a decline in egg production. Flocks were divided into two groups: Vaccinated and unvaccinated.

### Vaccination protocol

Vaccination against ILT in Algeria is reserved for valuable animals, such as grandparents, laying hens and breeders, and, sometimes, broilers. The vaccination protocol in Algeria is variable depending on the vaccine strain used in the field, so each laboratory suggests its own protocol (Table-1).

**Table-1:** ILT vaccine strains available on the field in Algeria.

Vaccine strain	Classification	Primo (Age by week)	Booster (Age by week)	Administration mode
CHP 50	Live attenuated CEO	2-3 weeks	10-16 weeks	Eye instillation
Hudson	Live attenuated CEO	4 weeks and more	desirable	Eye instillation or drinking water
Serva	Live attenuated CEO	4-6 weeks	14-16 weeks	Eye instillation
Vectored FP-LT	Vectored	8 weeks and more	/	Wing web

CEO=Chicken embryo origin, FP-LT=Fowl pox-laryngotracheitis, ILT=Infectious laryngotracheitis

Table-1 summarizes the strains available in the Algerian market. Three-quarters of the vaccines in the market are of chicken-egg origin (CEO), which carry the risk and problems of a return to virulence of the live attenuated vaccine. However, the only vector vaccine product available is expensive; hence, breeders opt for the cheapest method. The tissue culture origin (TCO) vaccine is not yet marketed in Algeria. The vector vaccine remains the best because it does not present any risk; however, the immune protection takes a long time to set in.

Of the 48 flocks selected for this study, 26 (54.2%) flocks were vaccinated for ILT using a live attenuated CEO vaccine; for the remaining 22 (45.8%) flocks, no vaccination was done against ILT.

### Clinical diagnosis

The presumptive clinical diagnosis was based on a clinical history provided by authorized farm staff, typically the veterinarians in charge of monitoring and recording the clinical signs, and gross lesions obtained by necropsy, which were a strong indication of ILT in affected chickens.

### Blood collection procedures

Two samples were collected from the selected hens from each farm according to the method described by Salhi *et al*. [10] and Messaï *et al*. [11]. The first was performed after the appearance of the first clinical signs. The second sample was collected after a time interval of 6-10 weeks, to allow for development of serum antibodies. A total of 1728 blood samples were collected from 48 flocks of layer hens (15-20 samples/flock) in dry tubes. The tubes were then centrifuged at 5000 rpm for 10 min on the same day to recover the sera, which were stored in Eppendorf tubes and frozen at −20°C until analysis.

### Serological tests

An indirect ELISA test kit (ID Screen^®^ ILT Indirect, IDvet, Montpellier, France) was used. ELISA is not the reference method for the diagnosis of ILT, but this technique has evolved, and the interpretation of results is also based on the baseline antibody titers, coefficient of variation (CV), serum positivity, and clinical lesions. In associating these different parameters, we can conclude whether there is an infection (viral passage of ILT), and we can also check if the herds are correctly vaccinated or not from the baseline titers of hens without clinical signs and without disease lesions.

The ELISA technique was carried out, as described in the literature [10,11]. Means of titers and the CV were automatically calculated using the software IDSoft™ 5.05 (Montpellier, France).

### Interpretation of the diagnostic results

Clinical signs, postmortem lesions, antibody kinetics, seropositivity, mean titers between the two sets of sampling, and the CV and ELISA baselines were all considered in interpreting the ELISA results. According to the baselines of IDvet, the expected average antibody titers after the first application of a live attenuated CEO vaccine vary from 1000 to 3000 within 6-10 weeks after vaccination, with 80-100% seropositivity; and after two vaccinations, the titers vary from 1000 to 4000. The CV must be between 40% and 60% to be considered a good vaccination. Titers below 1000 indicate a poor or no vaccination intake or an immuno-depressive disease, and more than 3000 for a single live vaccine, and 4000 for two live vaccines with a tight CV (<40%) indicate a viral passage.

### Observation of the risk factors

The standardized survey used to assess the risk factors associated with mortality and previously observed egg drop includes the following ten parameters: Season, climate (autumn, spring, and summer, along with the temperature variation from −5°C in winter to 45°C in summer), area, strains of layers, age of occurrence, density, hygiene, vaccination, mortality, and egg drop rate.

### Statistical analysis

First, SAS (Version 9.1.3; SAS Institute Inc., Cary, NC) was used for descriptive statistics to characterize flocks according to different factors. Before fitting the statistical analysis, examination of the distributions of antibody titers indicated using PROC UNIVARIATE and the Shapiro–Wilk test indicates that most of the data could not be considered normally distributed. If the variable does not fit the normal distribution, adjustments such as logarithmic, square, and square-root transformations are possible tools. Antibody titers of the disease over time were analyzed by fitting a mixed general linear model using the MIXED procedure of SAS to evaluate seropositivity between the first and second set of serum collection. Then, the effect of probability of seropositivity was assessed using mixed-effects multivariable models (PROC GENMOD), using a normal distribution and log it link functions, and flocks to model random effects. Variables used in the model included the different risk factors. Before including a mixed model, initial screening of variables was performed using a manual backward stepwise procedure with significant variables (p<0.1) remaining in the model. Finally, sensitivity and specificity of detecting diseases according to clinical and necropsic signs were calculated using the diagnostic test evaluation of Win Episcope 2.0 (WinEpi, Spain).

## Results

Table-2 presents the antibody titer scores for ILT. Of the 48 layer flocks, 27 (56.25%) were seropositive for ILT and have subsequently shown a low CV (13-49%) and a significant difference (p<0.0001) in the mean antibody average titer between the first and the second sets of samples (4135.00±278.19 vs. 11046.00±649.71).

**Table-2:** Serological scores of ILT among 48 layer hens flocks.

Pathology	Antibody titers		CV (%)	SE		p-value	Seropositivity (%)
	
	Mean 1	Mean 2		SE 1	SE 2		
ILT	4135.00	11046.00	13-49	278.19	649.71	<0.0001	56.25

ILT=Infectious laryngotracheitis, CV=Coefficient of variation, SE=Standard error

For the remaining 21 (43.75%) flocks, the ELISA scores were in the expected norms, and there were no signs or lesions that refer to ILT.

**Figure-2:**
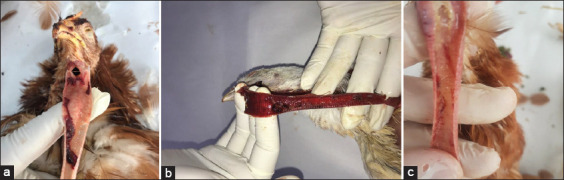
Gross pathologic tracheal lesions associated with various stage of laryngotracheitis infection in layer hens (a) hemorrhage in trachea, (b) fibrinohemorrhagic tracheitis, (c) caseous plug in trachea.

The ILT lesions and signs observed in the field included respiratory symptoms, such as sinusitis, conjunctivitis, hemorrhagic tracheitis, and various stages of laryngotracheitis infection (Figure-2), along with declines in egg production, high morbidity, and low mortality.

Our observations using necropsy and clinical signs to diagnose this disease were matched to our serological findings (Table-3), with 70% specificity. This means that all birds suspected of having ILT had specific antibodies. However, the sensitivity level was 55%. So, far, for this disease, clinical diagnosis, gross lesions, and ELISA testing were relatively reliable for diagnosing the disease.

**Table-3:** Diagnostic sensitivity (%) and specificity (%), with 95% CI and true prevalence of test based on lesional and clinical signs suggestive of ILT.

Pathology	Sensitivity (%) (95% CI)	Specificity (%) (95% CI)	True prevalence (%) (95% CI)
ILT	55.0 (45.5, 90.0)	70.4 (50.7, 100.0)	46.5 (36.8, 62.6)

CI=Confidence intervals, ILT=Infectious laryngotracheitis

Table-4 lists factors influencing the seropositivity of ILT. Flocks with bad hygiene had 68% higher seropositivity rates (odds ratio [OR]=1.68, p=0.002) compared to those with good hygiene. When vaccination was not applied, flocks were significantly more seropositive by 54% (OR=1.54, p=0.01) compared to vaccinated flocks. On the other hand, flocks with egg drop rates between 10% and 30% were significantly more seropositive by 42% (OR=1.42, p=0.04) than those flocks with egg drop rates >30%. However, there were no significant differences among the antibody titers for flocks based on the following parameters: Climate, season, area, age, density, layer strain, and mortality rate.

**Table-4:** Effects of different risk factors on the seropositivity for ILT on 48 layers flocks.

Factors	Value	Prevalence	Estimate	SE	OR	95% CI	p-value
Season	Autumn	14.1	0.20	0.17	0.79	0.56-1.11	0.60
	Spring	37.7	−0.37	0.21	1.06	0.78-1.68	0.18
	Summer	48.2			Ref		
Climate	Wet	68.6	−0.15	0.20	0.84	0.52-1.42	0.42
	Dry	31.4			Ref		
Area	West	54.3	−0.21	0.16	0.78	0.88-1.59	0.32
	East	21.5	0.51	0.19	0.96	0.69-1.89	0.78
	Center	24.2			Ref		
Strain	Hy-line	27.8	0.35	0.15	0.74	0.92-1.76	0.19
	Lohman	12.3	−0.07	0.24	1.38	0.38-1.47	0.67
	Tetra-SL	13.0	−0.09	0.31	0.88	1.09-2.7	0.77
	ISA Brown	46.9			Ref		
Age (week)	22-45	38.5	−0.02	0.15	0.90	0.77-1.37	0.89
	45-65	61.5			Ref		
Density (birds/cage)	>4	26.3	0.28	0.19	0.98	0.92-1.98	0.56
	≤4	73.7			Ref		
Hygiene	Bad	49.0	0.48	0.21	1.68	0.79-2.13	0.002*
	Intermediate	28.8	0.10	0.17	1.14	0.67-1.54	0.78
	Good	22.2			Ref		
Vaccination	No vaccine	45.8	0.37	0.24	1.54	0.89-2.32	0.01*
	Vaccine	54.2			Ref		
Mortality (%)	>5	21.2	0.12	0.18	1.06	0.57-1.65	0.21
	<5	78.8			Ref		
Egg dropping (%)	10-30	19.6	0.32	0.28	1.42	1.03-1.99	0.04*
	<10	27.3	−0.09	0.21	1.06	0.71-1.87	0.46
	>30	53.1			Ref		

ILT=Infectious laryngotracheitis, SE=Standard error, OR=Odds ratio, CI=Confidence interval,

* =significant difference, p-value of the risk factor in the same parameter

## Discussion

Clinical findings aid in diagnosing suspected ILT cases, but laboratory confirmation using histopathology is required [12,13]. This is because viral and bacterial poultry diseases with respiratory tropism have similar manifestations and symptoms and can therefore be clinically confused. Respiratory pathogens include the poxvirus, the wet or (diphteric) form of which affects the respiratory tree and the esophagus, manifesting by the pseudomembranes; *Mycoplasma gallisepticum*; Newcastle disease; avian influenza (H9N2); infectious bronchitis; and fowl adenovirus [4,13-15]. Among laboratory serology tests, ELISA is widely used to measure antibody titers. We have chosen the indirect ELISA screening method, which has proven to be the most practical serological test, as it is simple to perform, rapid, and requires very little serum [16-18].

Serology can detect infected birds but does not allow differentiation of carrier birds nor of vaccine and field strains. ELISA was found to be a sensitive method to measure anti-ILTV antibodies; making it the most often used method for its advantages, as reported by Garcia and Spatz [4].

In our study, the layer hen flocks chosen as samples had a seropositivity rate of 56.25% for ILTV, and a low CV (13-49%) has been shown, as well as a difference (p<0.0001) in mean antibody titers between the first and the second set of samples (4135.00±278.19 vs. 11046.00±649.71). A test result in the vaccinated flock is considered to be positive for ILT when sera tests are positive in both sets of blood samples, the antibody titers are much higher than the baseline vaccination titers (1000 and 4000), with very tight CV (<40%), and presenting mild signs and gross lesions, indicating viral spread. Indeed, as long as no vaccination against ILT is given, a positive serology result reveals circulation of the ILTV, where the titers are initially negative and become positive in the second set of sera samples; the seroconversion is associated of course with severe clinical signs, gross lesions, and decline in egg production.

Serologically, the elevation of antibody titers between the two sampling times indicates recent infection or symptomatic viral reactivation. On the one hand, the immune response is estimated by the level of specific antibodies produced against the wild virus or the vaccine strain. On the other hand, the protected flocks must have a high average of antibody titers for the baseline resulting from vaccination, with the absence of specific clinical signs [8,10,11,19].

In our study, observations of clinical signs and gross lesions of ILT included respiratory signs such as mucoid to hemorrhagic tracheitis, sinusitis, conjunctivitis, and egg production declines. ILT is not known to affect eggshell quality; some poultry houses affected by TRT (pneumovirus) exhibit pale eggshells, a high rate of morbidity, and low rate of mortality. Our observations are correlated with those reported by Garcia and Spatz [4], Jackwood and de Wit [14], Kirkpatrick *et al*. [20], Menendez *et al*. [21], and Kaboudi *et al*. [22].

For the factors affecting ILT, there was a significant effect of vaccination, hygiene, and rate of egg drop observed in the second set of samples on antibody titers. Unvaccinated layer flocks appeared to be more seropositive compared to vaccinated flocks by 54% (OR=1.54, p=0.01). Therefore, the mean antibody titers were 68% higher in flocks with bad hygiene than those with good hygiene (OR=1.68, p=0.002). At the least, flocks with egg drop rate between 10% and 30% were significantly more seropositive by 42% (OR=1.42, p=0.04). A seropositivity rate of 56.25% for ILT recorded in our study means that from 48 flocks, 27 were affected by ILT.

Several scenarios can explain the seropositivity rate of 56.25%. It can be due to an infection by the wild type ILTV, especially in unvaccinated flocks, or reactivation of the latent virus in birds that have recovered from ILT infection. The virus is latent in the trigeminal ganglia of recovered birds until its reactivation by stress, which leads to its excretion [4,23]. More likely, seropositivity is due to the circulation of reverted live attenuated CEO vaccine strains, caused by vaccine failure or inadequate vaccination practices.

Live vaccines protect against the ILTV [4]. In our study, 22 flocks were not vaccinated, and the remaining 26 flocks were vaccinated with live attenuated CEO vaccine, which means that five farms among the 26 vaccinated had probably sustained a viral passage despite the vaccination. Successful vaccination depends largely on the choice of vaccine strains and vaccination protocol [24-26]. Despite this, outbreaks of the disease in vaccinated flocks are known to occur quite frequently [24-26]. Outbreaks in the field are the consequence of circulating vaccine strains that have regained virulence due to the lack of biosecurity, inadequate vaccination, and latent carriers [4].

In Algeria, ILT vaccination is most often reserved only for valuable animals, such as layer hens and breeders; broilers are also sometimes vaccinated. However, three out of the four ILT vaccines used in Algeria are live strains (CHP50, Serva, Hudson), all of which are CEO; only one is a vector vaccine. In fact, CEO vaccines can propagate and recover their virulence after limited back passages between poultry farms [27-29]. Many molecular and epidemiological studies confirm that some outbreaks of ILT are caused by CEO vaccines, contrary to the TCO vaccine, which has a low ability to disseminate, and therefore less chance of regaining virulence [27-29]. The only TCO vaccine currently produced is the LT-Ivax; it is used only in the USA and Europe [24,25]. Vector vaccines do not recover their pathogenicity, do not spread, cannot be reactivated from latency, and cannot recombine to yield a virulent strain [4].

In Algeria, there is a lack of biosecurity measures, and separation between the different types of poultry production is rarely established. Very often, on the same poultry farm, we found two types of poultry being raised: Laying hens and broilers, or breeders and broilers. Broilers and unvaccinated layers are considered “naive chickens,” which can help and facilitate the transmission and reversal of the CEO vaccine strains, recovering their virulence and causing outbreaks. Furthermore, some broiler flocks are CEO vaccinated by group vaccination using coarse aerosol spray; post-vaccination reactions, spread, and reversion can occur, thus causing outbreaks [30].

The mean antibody titers in our study were higher in the flocks with bad hygiene than those with good hygiene, by 68% (OR=1.68, p=0.002). It should be stressed that no vaccine will solve the problem of disease if important health precautions are not taken. These include strict adherence to farming methods, cleaning, and disinfection of the premises, adherence to a sanitary vacuum practice compliance with the crawl space deadline, which must be at least 15 days, including hygiene of the livestock, feed, and housing, which together reduce the pressures of viral infection on a farm. Good hygiene and correct biosecurity measures are effective in preventing disease, thus reducing its economic impact [31,32].

Recent studies around the world (USA, Brazil, Norway, Palestine, Australia) show that ILT is responsible for egg-laying losses that exceed 30% in layer hens [33,34]. In our study, flocks with egg drop rate between 10% and 30% were significantly more seropositive, by 42% (OR=1.42, p=0.04). Jackwood and De Wit [14], Barhoom [33], and Parra *et al*. [34] reported that a moderate form of this disease is emerging in laying hen farms, and it manifests in very mild respiratory signs and a moderate drop in egg laying, between 10% and 15%, confusing ILT with other viral respiratory tropism diseases, especially infectious bronchitis. However, ILTV propagates slowly in a flock, but the clinical signs and gross lesions can be more severe [14].

ILT spread can be controlled by a correct vaccination program and strict biosecurity measures [4]. Furthermore, vaccination of poultry during an outbreak is known to be a good way to reduce the clinical manifestations, control infection strategies, and effectively limit the spread of the virus and shorten the course of disease [4]. In broilers, vaccination by drinking water is better than spray vaccination, to enhance flock coverage, prevent reactions, and avoid the spread and reversal of the CEO vaccine [35].

The prevention of disease is based on sanitary and medical prophylaxis, the control of which is first and foremost based on strict biosecurity measures. Thus, the most effective approach is to have quick laboratory results and implement a correct vaccination protocol to avoid the possible dissemination of viruses [36,37].

## Conclusion

The serological survey using ELISA revealed anti-ILTV antibodies, signifying the circulation of this virus in layer hen farms in Algeria, with a seroprevalence of 56.25%. In our study, risk factors such as poor hygiene, lack of biosecurity, and an inadequate vaccination program aggravate the disease, which can lead to huge economic losses in terms of production (declines in egg laying). Its diagnosis in the field is preliminary, and laboratory methods are useful for confirming the infection and making a correct diagnosis. A good vaccination protocol, including mass vaccination of layers, breeders, and broilers; the use of TCO and vector vaccines; strict biosecurity measures; and the rapid diagnosis and detection of latent carriers are necessary to eradicate the disease from our farms.

## Authors’ Contributions

OS, CRM, NO, IB, HK, RK, and DK designed all steps of the study. OS and CRM wrote the manuscript draft and collected all data. OS and CRM diagnosed the disease, OS, CRM, NO, IB, and HK collected the samples. OS, CRM, NO, IB, and HK carried out the laboratory work. OS performed statistical analysis. OS, CRM, KR, and DK analyzed and interpreted the data. OS, CRM, and DK reviewed the manuscript. All authors read and approved the final manuscript.
